# Hepatitis C Antibody Screening Among Baby Boomers by a Community-Based Health Insurance Company

**DOI:** 10.1089/pop.2020.0214

**Published:** 2021-08-16

**Authors:** John R. Litaker, Naomi Tamez, Carlos Lopez Bray, Robert D. Allison, Wesley Durkalski, Richard Taylor

**Affiliations:** ^1^ The Litaker Group, LLC, Office of Population Health and Science, Austin, Texas, USA.; ^2^ Sendero Health Plans, Austin, Texas, USA.; ^3^ University of Texas at Austin, School of Human Ecology, Public Health Program, Austin, Texas, USA.

**Keywords:** health education, hepatitis C virus, screening, Sendero Health Plans

## Abstract

Infection with the hepatitis C virus (HCV) is the most commonly reported bloodborne infection in the United States. Individuals born between 1945–1965, the baby boomers, account for approximately 75% of all chronic HCV infections in the United States. The purpose of this study was to determine if a 6-week intervention, including outreach, education, and incentive, by a community-based health insurance company could improve uptake of HCV antibody screening among the 1945–1965 birth cohort. Individuals were eligible to participate in this campaign if they were born on or after January 1, 1945 and on or before December 31, 1965, had health insurance with Sendero Health Plans during the intervention period, and had no evidence of having received an HCV antibody test prior to the campaign start date. The 6-week campaign period was from November 14, 2018 through December 31, 2018. A gift card incentive was provided if HCV screening was completed on or before December 31, 2018. A total of 5287 individuals were eligible to participate in the campaign. Members who were baby boomers were 3.36 times more likely to receive HCV antibody screening during the intervention period in 2018 than during a similar period in 2017 (prevalence ratio = 3.36; *P* < 0.0001; 95% confidence interval: 2.71, 4.16). Health officials have established the identification, treatment, and elimination of HCV as a national policy objective. Using an outreach, education, and incentive approach, Sendero Health Plans improved uptake of HCV antibody screening among the high-risk baby boomer population.

## Introduction

Infection with the hepatitis C virus (HCV) is the most commonly reported bloodborne infection in the United States.^1^ An estimated 2.1 to 2.4 million noninstitutionalized US adults are chronically infected.^1,2^ People born between 1945–1965, the baby boomers, are 5 times more likely to have chronic HCV infection as compared to other adults. Baby boomers account for approximately 75% of all chronic HCV infections in the United States.^3,4^ Persons who inject drugs and pregnant women represent 2 cohorts with increasing incidence of HCV infection.^5^ The most significant consequences of chronic HCV infection include liver cirrhosis, hepatocellular carcinoma, and death.

At the time of this study the US Centers for Disease Control and Prevention (CDC) and the US Preventive Services Task Force (USPSTF) recommended 1-time screening for individuals in the 1945–1965 birth year cohort.^6,7^ Currently, the USPSTF recommends all adults aged 18 to 79 years receive an anti-HCV antibody test.^8^ Individuals younger than age 18 and ∼79 also are recommended for screening if they are at high risk for HCV infection.^8^ All persons who screen positive for the HCV antibody will undergo HCV ribonucleic acid testing to determine HCV chronicity status. The ability to identify chronic HCV status can lead to treatment with direct-acting antivirals, which are shown to have a >95% sustained virologic response with few short-term side effects.^9^ The revised 2020 USPSTF recommendations for HCV screening indicate a benefit to screening older populations as they also can benefit from using direct-acting antiviral medications.^5^

In 2018, Sendero Health Plans, Inc. (Sendero), a community-based, nonprofit health maintenance organization in Austin, Texas providing health insurance coverage under the Patient Protection and Affordable Care Act (ACA), designed and assessed a brief HCV screening campaign to increase HCV antibody screening among individuals in the baby boomer cohort. Sendero has a statutory obligation under ACA legislation to provide coverage for USPSTF items with a recommendation rating of “A” or “B,” which includes HCV antibody screening.^10^ Herein are presented the results of a pilot study that used outreach, education, and financial incentive to encourage individuals in this at-risk group to obtain the recommended 1-time HCV antibody screening test.

## Methods

Individuals were eligible to participate in this campaign if they were born on or after January 1, 1945 and on or before December 31, 1965 (ie, baby boomers) without a previously documented HCV antibody test as evidenced by Current Procedural Terminology (CPT) codes: 86804 (confirmation of HCV antibody testing); 86803 (HCV antibody screening); 87902 (HCV genotyping); or 87521 (HCV quantification) in the Sendero claims database. The baby boomer cohort was selected because (1) individuals born in this cohort account for 75% of all chronic cases of HCV infection; (2) the CDC and USPSTF had specific guidelines and recommendations related to antibody screening for this cohort at the time this study was completed; and (3) date of birth can be used with confidence to identify individuals in this cohort from the Sendero claims system.

Individuals eligible to participate in this campaign were sent an outreach letter and educational material on November 14, 2018. The outreach letter contained information on the screening campaign and information about treatment options. Educational material from the CDC was provided.^11^ Members were informed that there were no out-of-pocket costs for the provider visit or for the screening test. Individuals who obtained the HCV antibody test on or before December 31, 2018 were told that they would receive a $50 gift card. Follow-up text and automated phone calls (robocalls) were conducted throughout the campaign. Information was provided in both English and Spanish.

Providers had until 90 calendar days after service delivery to submit a claim; therefore, ongoing review of claims submissions occurred through March 31, 2019. After March 31, 2019, all eligible members who had a claim for CPT code 86803 performed from November 14, 2018 through December 31, 2018 were deemed to have participated in this campaign and were mailed a $50 gift card.

The outcome of interest for this intervention was receipt of an HCV antibody test by eligible members of the baby boomer birth cohort. Results from this campaign period were compared to a comparison period of the baby boomer population from a year earlier (November 14, 2017–December 31, 2017), during which no education, outreach, or incentive was provided. A chi-square test of independence was used to evaluate the intervention. Descriptive statistics are also reported.

This project was approved as a quality improvement initiative by Sendero Health Plans on September 1, 2018. Sendero received approval from the Aspire Institutional Review Board to conduct secondary data analysis on claims related to this quality improvement initiative (Protocol number 2016F-09-01, December 4, 2018).

## Results

A total of 5287 persons were eligible to participate in this campaign. Among them, 316 (6.0%) obtained an HCV antibody test during the intervention period of November 14, 2018–December 31, 2018. Females accounted for 181 (57%) screenings during the intervention period and males accounted for 135 (43%) screenings. Females were 8% more likely to be screened than males, although this was not statistically significant ($x^{2}$ [1] = 1.08, *P* = 0.41). Of the 316 people screened during the 2018 intervention period, there were 31 pairs of members (n = 62) living in the same household.

Further analyses compared the Sendero baby boomers eligible for screening during the 2018 intervention period to the Sendero members eligible for screening during a similar period during 2017 (a comparison period). In calendar years 2017 and 2018, claims data indicates that 795 and 840 eligible baby boomer members obtained an HCV antibody test, respectively (Table 1). Eighty-nine of 795 (11.2%) baby boomers obtained the HCV antibody test during the 2017 comparison period, while 316 of 840 (37.6%) baby boomers obtained the HCV antibody test during the 2018 intervention period. Baby boomers were 3.36 times more likely to be screened during the 2018 intervention period as compared to a similar period during 2017, when no intervention occurred (prevalence ratio = 3.36; *P* < 0.0001; 95% confidence interval: 2.71–4.16).

**Table 1.** Count of Screening Hepatitis C Virus Antibody Encounter Data for the Study Cohort in Calendar Year 2017 and Calendar Year 2018, by Month (*χ*^2^ [23] = 88.80, *P* < 0.001)

Year	Jan	Feb	Mar	Apr	May	Jun	Jul	Aug	Sep	Oct	Nov	Dec	Total
2017	50	71	78	59	72	48	57	87	76	75	70	52	795
2018	57	39	69	48	57	43	35	48	49	63	91	241	840

## Discussion

Chronic HCV infection may progress to liver cirrhosis, end-stage liver disease, and hepatocellular carcinoma, with late diagnosis associated with more severe outcomes, including death.^12^ A majority of acute HCV infections are asymptomatic, and about half of all chronically infected individuals are unaware of their infection status.^13^ Antiviral regimens for HCV are well tolerated and result in a >90% cure rate.^14^ Identifying cases using antibody screening, therefore, is an important strategy to identify, treat, and eliminate HCV in the general population. Currently, most HCV screening occurs secondary to a non-HCV presenting complaint at the point of entry to the health care system, including at clinics, emergency departments, and at inpatient admission.^15–18^ Although these settings represent an opportunity to screen for HCV infection, more needs to be done to identify individuals with chronic HCV infection who, if left untreated, might suffer HCV-related morbidities or mortality.^19^

There are multiple levels at which interventions could occur to improve uptake of HCV screening, including at the payer level. As a health insurance company, Sendero has a statutory obligation to provide screening tests that meet USPSTF “A” and “B” recommendations. As a community health plan established by the taxpayers of Travis County, Sendero also is socially accountable to its members to provide local solutions to support the national agenda. In this case, the national agenda has identified HCV identification, treatment, and elimination as a policy goal.

In this study the authors hypothesized that if the payer (Sendero) provided the opportunity and incentive, a member of a community-based health plan would opt to be screened for HCV. Results of this pilot project demonstrated that when outreach, education, and financial incentive are provided, individuals at risk for a disease will get screened absent another reason to visit a health care provider. With 6.0% of the eligible cohort screened during the 6-week intervention period, this study demonstrated that a community-based health insurance plan can play a role in strengthening the public health response to HCV screening. Furthermore, by addressing this cohort of individuals now, the burden of outreach, education, and treatment is shifted away from the public health system, allowing public health officials to concentrate screening efforts on individuals who do not have health insurance and who, therefore, do not already have a point of entry into the health care system.

With regard to the financial incentive component of the intervention, there may be some disquiet about whether it is appropriate to use financial incentives as a policy instrument to increase uptake of HCV antibody screening. The economic and ethical rationale for financial incentives to support uptake of medical services is well established.^20–22^ Economically, a modest incentive, if successful in encouraging individuals to undertake a health benefit, can reduce downstream spending on inpatient and outpatient medical care. Ethically, because the decision to participate in the incentive program is voluntary, a member can either participate or not participate. Indeed, 4971 individuals chose not to participate in this campaign.

With the success of this pilot project there may be opportunities to identify other levels in the health care system in which outreach, education, and financial incentive could be provided in order to screen for HCV. However, depending on the target population, the payer may wish to consider targeting the provider instead of the member. For example, it would be unlikely that the payer could identify persons who inject drugs based on CPT or International Statistical Classification of Diseases and Related Health Problems, Tenth Revision (ICD-10) claims data, particularly if such persons have not previously presented with a condition indicative of injection drug use. As such, the payer could work with providers to identify and screen persons who inject drugs in support of the USPSTF recommendation and provide the provider an incentive payment when such screening occurs. This would be consistent with pay-for-performance activities that align payment policies with quality improvement initiatives.^23^ Other at-risk populations (eg, pregnant women) could be targeted directly by the payer with outreach, education, and incentive once a pregnancy-related CPT or ICD-10 claim is filed.

This study has limitations. Firstly, the study period was brief and included 3 major US holidays, thus potentially reducing an individual's ability to either schedule an appointment or complete HCV screening by December 31, 2018. Secondly, the authors do not know the extent to which each component of the cue to action was most responsible for a person's decision to obtain the exam. Previous research by this community-based health plan showed that financial incentives alone and educational outreach alone were equally effective in increasing uptake of a medical eye examination among individuals diagnosed with diabetes.^24^ Thirdly, the ideal study design for this intervention would have been a randomized controlled trial. However, because this intervention was a USPSTF recommendation, the goal was to try to maximize the number of people who would respond to this cue to action so that any positive cases identified could be referred to treatment. Finally, further research is needed to understand if additional interventions (eg, transportation assistance) could further improve HCV screening in this population or if similar findings could be demonstrated if a broader population (eg, individuals aged 18–79 years) was incentivized, as identified by the new USPSTF guidelines.^8^

## Conclusion

The national agenda has established the identification, treatment, and elimination of HCV as a national policy objective. Implementation of policy, however, occurs at the local level. Multiple organizations have a role in implementing policy at the local level, including a health insurance company. Using an outreach, education, and incentive approach, Sendero Health Plans improved uptake of HCV antibody screening by 6.0% (prevalence ratio: 3.36; *P* < 0.0001; 95% confidence interval: 2.71–4.16) compared to a similar period of a year earlier without an intervention among the high-risk baby boomer population, in accordance with the national agenda.

## Author Contribution Statement

Drs. Litaker, Allison, and Taylor, and Ms. Tamez, Mr. Bray, and Mr. Durkalski have met the following contribution requirements: (1) substantial contributions to the conception or design of the work; or the acquisition, analysis, or interpretation of data for the work; AND (2) drafting the work or revising it critically for important intellectual content; AND (3) final approval of the version to be published; AND (4) agreement to be accountable for all aspects of the work in ensuring that questions related to the accuracy or integrity of any part of the work are appropriately investigated and resolved.
